# 

**Published:** 1881-03

**Authors:** 


					﻿Whole Number,
CCCCXXXV.
March, 1881.
Number 3,
Vol. XLI1.
THE
CHICAGO
M E D I C A L
T ournal and Examiner
EDITORS:
N. S. DAVIS, M.D., LL.D. JAS. NEVINS HYDE, A.M., M.D.
DANIEL R. BROWER, M.D.
CHICAGO:
PUBLISHED BY THE CHICAGO MEDICAL PRESS ASSOCIATION,
188 Clark Street.
jgF'Read the Notice of this Journal among Advertisements.
				

